# Bovine Serum Albumin Nanoparticle-Mediated Delivery of Ribavirin and Mycophenolic Acid for Enhanced Antiviral Therapeutics

**DOI:** 10.3390/v17020138

**Published:** 2025-01-21

**Authors:** Mayra A. Castañeda Cataña, Andrea P. Rivas Marquina, Martín M. Dodes Traian, M. Josefina Carlucci, Elsa B. Damonte, Oscar E. Pérez, Eva C. Arrua, Claudia S. Sepúlveda

**Affiliations:** 1 Instituto de Química Biológica de la Facultad de Ciencias Exactas y Naturales (IQUIBICEN), UBA-CONICET, Buenos Aires 1428, Argentina; macc@qb.fcen.uba.ar (M.A.C.C.); mdodestraian@qb.fcen.uba.ar (M.M.D.T.); majoc@qb.fcen.uba.ar (M.J.C.); edamonte@qb.fcen.uba.ar (E.B.D.); oscarperez@qb.fcen.uba.ar (O.E.P.); 2 Centro de Investigación y Desarrollo en Materiales Avanzados y Almacenamiento de Energía de Jujuy-CIDMEJu (CONICET-Universidad Nacional de Jujuy), Centro de Desarrollo Tecnológico General Savio, Buenos Aires 4612, Argentina; arivas@cidmeju.unju.edu.ar (A.P.R.M.);; 3 Laboratorio de Estrategias Antivirales, Departamento de Química Biológica, Facultad de Ciencias Exactas y Naturales, Universidad de Buenos Aires (UBA), Buenos Aires 1428, Argentina

**Keywords:** mycophenolic acid, ribavirin, albumin nanoparticles, Zika virus, Junín virus, vesicular stomatitis virus, herpes simplex virus

## Abstract

The global spread of viral diseases is a public health issue. Ribavirin (RBV) and mycophenolic acid (MPA) are well-known wide-spectrum antiviral agents. The present study evaluated the potential of bovine serum albumin (BSA) nanoparticles (NPs) as a vehicle to improve the efficacy of molecules with antiviral activity. The results demonstrated that NPs offer a promising strategy for the delivery of antiviral drugs, improving their stability and reducing toxicity compared to free agents. BSA-based NPs effectively encapsulated hydrophilic molecules such as MPA and water-soluble compounds such as RBV, achieving encapsulation efficiencies of 10% and 20%, respectively. The purified NPs exhibited a particle size between 60 and 100 nm and did not show toxicity at the evaluated concentrations. In cellular viral infection models against Zika virus (ZIKV), Junín virus (JUNV), vesicular stomatitis virus (VSV) and herpes simplex virus (HSV-1), the BSA-based NPs loaded with MPA or RBV demonstrated antiviral properties superior to those of non-encapsulated agents, as well as 100- and 200-fold effective dose reductions, respectively. These findings clearly indicate the potential of BSA NPs as a novel platform for the development of safer and more efficient antiviral therapies.

## 1. Introduction

The continuous emergence or reemergence of viruses in recent decades has occasionally led to outbreaks or global pandemics, posing a significant challenge to public health. This underscores the urgent need for the development of effective antiviral agents [1]. No specific chemotherapy or safe vaccination protocols are currently available for several viral human pathogens. Antiviral therapy has traditionally relied on drugs that target specific viral proteins, often with a narrow viral spectrum of effectiveness. However, this approach is constrained by the rapid mutation rate of some viruses, particularly RNA viruses, which can readily evolve resistance to therapies. In response to these limitations, research has increasingly focused on the development of host-targeting antivirals (HTAs) directed towards the cellular processes involved in viral replication. HTA are typically broad-spectrum antivirals and can prevent the development of resistance mechanisms that arise from the selection of viral mutants [2,3,4,5,6,7]. For example, the specific MEK1/2-inhibitor Zapnometinib (ZMN) [8], the chemokine receptor CCR5 targeted Maraviroc [9], the alpha-glucosidase I inhibitor Celgosivir [10] and many others [11] affect mechanisms essential for viral replication that cannot be compensated by the virus. Furthermore, therapeutics targeting host factors hold promise for combating newly emerged viruses before in-depth characterization of the virus and enhances the possible prevention of outbreaks before escalating into global pandemics. Additionally, many of these drugs are repurposed from existing medications used for other therapeutic purposes, offering the advantage of a well-established safety profile and a reduction in the costs associated with bringing new drugs to the market [12,13].

In this context, inosine monophosphate dehydrogenase (IMPDH), the enzyme responsible for a rate-limiting step in the de novo intracellular synthesis of guanosine nucleotides, has been widely studied as a promising HTA [14,15]. Mycophenolic acid (MPA) and ribavirin (RBV) are known potent inhibitors of this enzyme [15]. MPA is a non-competitive inhibitor of IMPDH with antiproliferative, immunosuppressive and antiviral properties [6]. As for RVB, it is a nucleoside analog that can be phosphorylated inside the cell and acts as a competitive inhibitor of IMPDH [16]. Several mechanisms have been proposed for the antiviral action of RBV against RNA viruses [17,18,19,20]. Depending on the virus–cell system, the mode of inhibition exerted may vary, with one or more of the mechanisms predominating. RBV is currently an approved therapy for the treatment of hepatitis C and certain viral hemorrhagic fevers [13], whereas the use of MPA as an antiviral drug has not been approved by any health regulatory agency.

A wide range of antiviral substances have been reported to inhibit in vitro viral infections [17]; however, antiviral agents may confront significant challenges and often have a limited efficacy within in vivo administration due to their low solubility, instability during storage or application, low bioavailability, potential to cause adverse side effects or toxicity and the ability to select resistant viral variants [18]. On the other hand, the drug delivery method may introduce an additional barrier. Thus, oral administration is subject to enzymatic degradation and the acidic pH of the human gastrointestinal tract, which can compromise the chemical stability and structural integrity of the drug, resulting in a reduced bioavailability and poor therapeutic efficacy [19]. Low permeability through biological barriers such as the epidermis, mucosal layers and cell membranes, along with rapid digestion and accelerated elimination of metabolites, represent significant obstacles to the efficacy of various compounds [20,21], limiting their absorption, distribution and retention in the body.

Bovine serum albumin (BSA)-based nanoparticles (NPs) have gained significant interest in recent years due to their potential to improve the efficacy of traditional antiviral treatments [22]. BSA, a natural protein abundant in mammalian serum [23], offers a promising platform for the targeted delivery of antiviral agents [24], due to its distinct physical and chemical characteristics and its compatibility with cells and tissues [25,26,27]. It exhibits a high solubility, stability in the pH range of 4 to 9 and an alpha–helical secondary structure at room temperature and it is often used as a protein model for charged NP interactions or drug delivery. Protein NPs could encapsulate and transport bioactive substances and drugs [28].

Desolvation, or coacervation, is a common and easy albumin NP production process [29,30]. This approach requires adding ethanol or acetone to the albumin aqueous solution with agitation until it becomes turbid [31]. For the optimal albumin NP size, the flow rate and volume of the desolvating agent are crucial, as ethanol influences protein structure through solvent dielectric constant, solvation forces, hydrophobic interactions, hydrogen bonding, dipolar moments, and salt bridging [32]. Water-based solutions become less polar when alcohols are added, strengthening hydrogen bonding and α-helical structures while reducing hydrophobic interactions [30].

The design and engineering of NPs allow for precise control over their size, surface charge, and drug-loading capacity, enabling tailored drug delivery strategies for specific viral infections [33]. Their ability to circulate in the bloodstream for extended periods enhances drug exposure at the site of infection, thereby increasing antiviral activity [21]. Additionally, the fact that it is a natural protein reduces the likelihood of immune responses or allergic reactions, making these NPs suitable for sustained administration to treat chronic viral diseases [34].

The primary objective of our innovative study was to utilize the desolvation method to encapsulate hydrophilic drugs, such as RBV, and hydrophobic drugs, such as MPA, into albumin NPs. We then conducted a thorough physicochemical characterization of the BSA NPs and evaluated their biological activity against in vitro viral infection models. With this study, we aim to demonstrate the feasibility of this drug delivery system and its potential to optimize the treatment of infectious diseases.

## 2. Materials and Methods

### 2.1. Reagents

All reagents and solvents were analytical grade (Merck KGaA, Darmstadt, Germany). BSA (purity > 96%) and RBV (purity ≥ 98%) were acquired from Sigma-Aldrich (St. Louis, MO, USA). All solutions were produced using 18 MΩ ultrapure water (Milli-Q system, Merck-Millipore, Darmstadt, Germany). Novartis Argentina provided Myfortic^®^ 360 mg and from this commercial formulation, the excipients were eliminated through crystallization strategies, resulting in an MPA with 80% purity, which was subsequently characterized by NMR spectroscopy [35].

### 2.2. Preparation of NPs

BSA NPs were obtained using the desolvation method described by Díaz Saldívar and Huidobro-Toro [31] with modifications and under sterile conditions. To begin with, 1 g of BSA powder was dissolved in ultrapure water until it reached 1% wt/wt, adjusting the pH to 5–5.5 with 0.1 N NaOH solution and stirring overnight for complete protein hydration. Subsequently, the BSA solution was divided into 3 aliquots, with 1 aliquot containing BSA alone (BSA-NP), another with 100 µM of the active substance (AS) MPA (MPA@BSA-NP) and the last with 100 µM of the AS RBV (RBV@BSA-NP). Each mixture was stirred continuously at 150 rpm at 25 °C overnight. NP formation was induced by the dropwise addition of absolute ethanol (EtOH) at a rate of 1 mL/min and stirring until the solution became translucent [36] (considered a colloidal suspension). The suspension was ultracentrifuged at 20,000× *g* for 1 h at 4 °C (Class S XL-90 Beckman, Indianapolis, IN, USA), and the supernatant was discarded. The isolated NP pellet was suspended in ultrapure water, and this process was repeated three times. Finally, the pellet was redispersed in 5 mL ultrapure water (considered as purified NP). Each dispersion material was sonicated (Qsonica, Newtown, CT, USA) for 1 min.

### 2.3. Determination of Particle Size and Zeta Potential

The NP particle size was determined by photon correlation spectroscopy. The samples were prepared by ten-fold dilution of the NP suspension with distilled water using a polystyrene cuvette. Zeta potential (ζ-P) was determined according to the electrophoretic mobility of the NPs at 25 °C, using a SZ-100 Horiba and DTS1070 cuvette (HORIBA Instruments Inc., Irvine, CA, USA) equipment described by Arrua et al. [37]. Measurements were performed in triplicate and the results are expressed as the mean ± SD.

### 2.4. Scanning Electron Microscopy

The NP suspension was dried using vacuum centrifugation (Savant™ SPD131DDA, Thermo Fisher Scientific Inc., Waltham, MA, USA). After that, the dehydrated NPs were covered with a 10 nm gold layer. The particle size and distribution were examined from images obtained by Scanning Electron Microscopy (SEM) (Carl Zeiss NTS SUPRA 40, Germany), under an accelerating voltage of 10 kV, with a 3.0 nm resolution at high voltage and a 4.0 nm resolution at low voltage. The monochrome images of the BSA NPs were performed using the ImageJ 1.54f open-source software.

### 2.5. Stability Test

The stability of the nanosystem was assessed under various settings, including an NP colloidal suspension, purified NPs, frozen/thawed NPs, vacuum-dried NPs, and NPs kept at roughly −80 °C and 25 °C. The colloidal suspension and purified NPs were maintained at temperatures of 25 °C and −80 °C, respectively, for three months. The DLS approach was employed, as previously described, to assess the particle size (Z-ave), ζ-P and polydispersity index (PdI).

### 2.6. Encapsulation Efficiency

To determine the encapsulation efficiency (EʄE) of MPA and RBV into NPs, 2 mg of each sample was dissolved in 2 mL of trypsin (50 μg/mL). The solutions were stirred overnight at 500 rpm [21]. Then, the samples were centrifuged (5810R Eppendorf, Hamburg, Germany) for 20 min at 20,000× *g* at 4 °C. Finally, an aliquot of this suspension (500 µL) was diluted with ultrapure water up to a final volume of 1 mL. Empty NPs were treated in the same way to rule out any interference.

The RBV concentration was determined by UV-VIS spectrophotometry at λ = 210 nm (V-650 JASCO, Spectrophotometer, Japan) at 25 °C using a calibration curve in the range of 5–200 µM in water (R^2^ > 0.9997) using the protocol described in [38]. The MPA concentration was determined by fluorescent spectrophotometry (Aminco-Bowman Serie 2, Thermo Fisher Scientific Inc., USA) excited at λ = 342 nm with an 8 nm band pass, and the emission was read at λ = 425 nm at a constant voltage that was set between 700 and 900 V, and interpolating the measurements into a calibration curve of fluorescence intensity at MPA concentrations which ranged between 0.3 and 100 µM in water (R^2^ > 0.9997), as described in [39].

The encapsulation efficiency (%EʄE) of the particles was calculated according to Equation (1) previously described [35].

EʄE % = [(MPA or RBV initial) − (Free MPA or RBV)]/(MPA or RBV initial) × 100(1)

where MPA/RBV initial is the quantity put into the system to be encapsulated into NPs, and free MPA or RBV is the experimental nonloading antivirals. We reported the data as a percentage of the encapsulation efficiency. Results are reported as mean ± standard deviation (SD) from triplicate measurements.

### 2.7. Cell Cultures and Viruses

Vero cells, derived from African green monkey kidney (ATCC CCL-81), A549 cells, from human lung adenocarcinoma (ATCC CCL-185), and Huh-7 cells, from human hepatoma (RRID: CVCL_0336), were grown as monolayers in Eagle’s minimum essential medium (MEM) (Gibco, Thermo Fisher Scientific Inc., USA) containing 5% inactivated bovine serum and 50 µg/mL gentamycin. The serum concentration was reduced to 1.5% in the maintenance medium (MM). The Zika virus (ZIKV) strain PRVABC59 (ATCC #VR-1843DQ) [40], Junín virus (JUNV) strain IV4454 [41], herpes simplex 1 virus (HSV-1) strain KOS (ATCC #VR-1493), generously supplied by Dr. Erik De Clercq (Rega Institute, Leuven Belgium), and the vesicular stomatitis virus (VSV) strain Indiana Lab [V-520-001-522] were employed for the antiviral assays.

### 2.8. Cytotoxicity Assay

The 3-(4,5-dimethylthiazol-2-yl)-2,5-diphenyl tetrazolium bromide (MTT, Sigma-Aldrich, St. Louis, MO, USA) method [42] was used to measure cytotoxicity. Vero, Huh-7 and A549 cells were seeded at a density of 1.5 × 10^4^ cells per well in 96-well microplates using 100 μL of MEM and then incubated overnight at 37 °C in a 5% CO_2_ atmosphere. Thereafter, monolayers were covered with serial dilutions of AS or NPs in MM and incubated for 96 h at 37 °C until the MTT assay was performed. Absorbance at 570 nm was measured with a multi-mode microplate reader (FLUOstar^®^ Omega, LABTECH, Germany).

The cytotoxic concentration 50% (CC_50_) was defined as the concentration of AS or NPs needed to kill 50% of the cell population and was determined as the compound concentration required to reduce the MTT signal by 50% compared to untreated controls using Equation (2). Additionally, a blank sample blank consisting of cells cultured in a medium without NP or antiviral agents was used as a reference to assess antiviral activity in the experimental groups. Blank samples are used to zero an instrument during testing. Before adding chemicals, a blank sample can compensate for color or turbidity errors. Measurements were performed in triplicate and the results are expressed as the mean ± SD.

Cellular Viability % = [(S.T. _Abs_) *−* (S. Blank _Abs_)/(S. Unt. _Abs_) − (S. Blank _Abs_)] × 100 (2)

where

S.T.: sample treated; S. Blank: sample blank; S. Unt.: sample untreated

### 2.9. Plaque Assay

Vero cells grew overnight in 24-well microplates. Ten-fold dilutions of the virus samples were applied to cell monolayers and incubated at 37 °C for 1 h. Then, inocula were removed and monolayers were covered with MM containing 1% methylcellulose. Cells were incubated at 37 °C until lysis plaques were observed in each individual viral strain, then fixed with 10% formaldehyde, washed and stained with 1% crystal violet in 10% acetic acid [43], and viral lysis plaques were counted. The viral titer was expressed in plaque-forming units per milliliter (PFU/mL).

### 2.10. Antiviral Assay

The virus yield inhibition assay was employed to evaluate antiviral activity. Vero, A549 and Huh-7 cells cultured in 24-well microplates were infected at a multiplicity of infection (MOI) of 0.1 PFU/cell of each virus. After 1 h of incubation at 37 °C, the inocula were removed and cells were covered with MM containing or not containing (VC) serial dilutions of free AS or NPs. After 48 h at 37 °C, supernatants were collected, and viral yields were titrated using the plaque assay (2.9). The inhibitory concentration 50% (IC_50_) was calculated as the concentration required to reduce virus yield by 50% in the compound treated cultures, compared with untreated ones, using Equation (3).

Viral inhibition % = [100 − (viral titer)/(viral control titer)] × 100(3)

### 2.11. Statistical Analysis

Statistical analysis was performed using GraphPad Prism 8 (San Diego, CA, USA). The results were compared using one-way ANOVA followed by a post hoc test with Dunnett’s correction. To indicate statistical significance, the following cutoffs were used: * *p* < 0.05, ** *p* < 0.01 and *** *p* < 0.001. All tests were conducted in triplicate and the data were expressed as the mean ± SD.

## 3. Results and Discussion

### 3.1. Determination of Particle Size and Size Distribution

**Figure 1:**
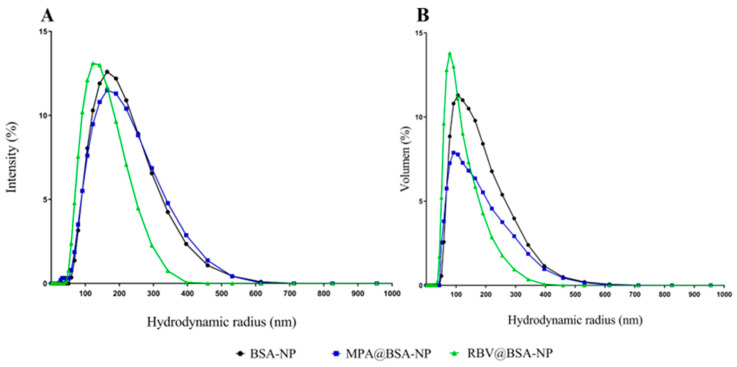
Hydrodynamic radius distribution of the NP colloidal suspensions. The graph in (**A**) displays the intensity distribution, whereas (**B**) illustrates the volume distribution.

The colloidal and purified NP suspensions were analyzed using DLS. The results for the particle size distribution of the colloidal suspension are shown in Figure 1 for BSA-NP without any AS as a control and for MPA@BSA-NP and RBV@BSA-NP. The distributions showed a monomodal pattern, with no substantial differences when the results were analyzed using intensity or volume. Parameters derived from particle size distribution are summarized in Table 1, which contains the values of Z-ave, PdI and ζ-P.

Optimal conditions for the production of NPs, such as pH, the infusion rate and the concentration of the desolvation agent, could alter the solubility of the protein and thus the NP aggregation in the solution [22,31]. The influence of MPA and RBV on the size of the colloidal suspensions and purified NPs was evaluated. The colloidal suspensions had a hydrodynamic radius of 120–155 nm, a PdI indicating monodispersity and ζ-P of 6–8 mV (Table 1). In the case of purified NPs, the hydrodynamic radius was located in the range of 56–74 nm, the PdI indicated monodispersing and there was an increased ζ-P with respect to the colloidal suspension (Table 1).

The viability of NPs for a certain method of drug delivery relies on several factors, including their average diameter, PdI and size stability [44]. Ensuring control and verification of these characteristics is crucial for the successful clinical use of nanocarrier formulations. For this kind of NP, PdI values ≤ 0.2 are most commonly deemed acceptable. Although the last edition of the FDA’s “Guidance for Industry” emphasizes the importance of size and size distribution as “critical quality attributes (CQAs)”, as well as essential components of stability studies of these products, it does not mention the criteria for an acceptable PdI [45].

In general, the formation of NPs from an AS solution occurs in three steps: nucleation, growth, and aggregation. Although the BSA-NP’s size is within the optimal range for biological applications, the particles’ loading capacity might be constrained by their minuscule size [46,47].

### 3.2. Encapsulation Efficiency

NP encapsulation efficiency (EʄE) is essential for improving drug delivery systems, targeting delivery and enhancing therapeutic efficacy while reducing adverse effects. The EʄE was measured in the supernatant after degradation of the NP with trypsin, using fluorescence spectroscopy for MPA and absorbance for RBV. The NPs exhibited an EʄE of 10.45% for MPA and 20.08% for RBV. The minimum content of both AS was in the µg range. In the literature, it is commonly reported in the order of mg [21,34,36], and it should be noted that reports on BSA NP with antiviral agents such as MPA or RBV are limited, except for our previous work [35].

The encapsulation efficiency was directly influenced by the concentration of AS added. It is necessary to explore if it is feasible to increase the concentrations of AS, and if this translates into EʄE values higher than those reported here. Notably, the physicochemical interactions during the preparation of BSA based NP did not influence the biological impact of the AS. There may be a limit to the amount of medicine that can be integrated within the BSA-NP due to its small size (56–74 nm) and the limited surface area available for encapsulation.

### 3.3. Scanning Electron Microscopy

**Figure 2:**
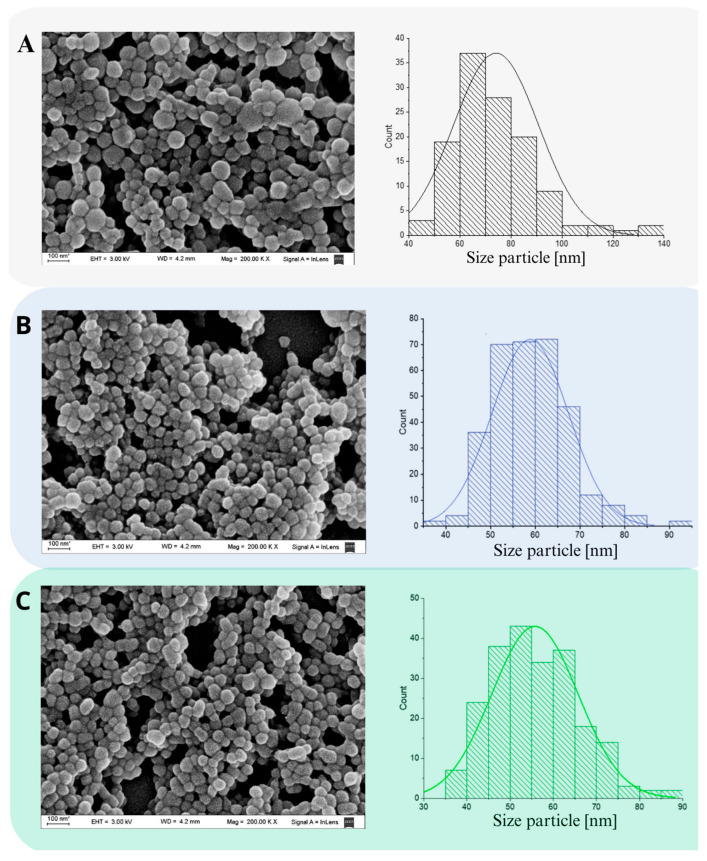
SEM images of NP. (**A**) BSA-NP; (**B**) MPA@BSA-NP; (**C**) RBV@BSA-NP. The histograms presented depict the size distributions of the respective micrographs. The data displayed represent the mean ± SD of n = 3 independent batches of NP.

SEM is a useful technique for monitoring morphological changes in preparation systems and surfaces after isolation and purification. Figure 2 shows NP with well-defined spherical shapes and porosities, obvious edges and no fractures (Figure 2A–C), with a Z-ave of 74.00 ± 2.00 nm, 59.00 ± 8 nm and 56.00 ± 10 nm, for BSA-NP, MPA@BSA-NP and RBV@BSA-NP. The distribution was unimodal for BSA-NP and MPA@BSA-NP (Figure 2A,B), while the histogram of RBV@BSA-NP indicates a bimodal population (Figure 2C). It was reported that the characteristics of size, charge, surface chemistry, shape and structure of NPs directly affect the way in which they interact with the biological environment and, ultimately, determine the associated cytotoxicity potential [48].

The inclusion of AS resulted in a decrease in a size decrease of the NP, suggesting a phenomenon of uniform nucleation. Supersaturation, which precedes nucleation, is essential to achieve a high nucleation rate, ensuring proper separation between nucleation and growth phases [22,49,50]. This process is crucial for the formation of uniform particles, as it prevents an excessive overlap between these phases and reduces the likelihood of aggregation, coagulation and Ostwald ripening [37,51].

### 3.4. Stability Test

Examining the colloidal and structural stability of NPs provides fundamental information about their structure. The response of NPs to storage conditions, for instance, the effect of the environment, is crucial for their use in in vitro models and for generating interest in the pharmaceutical industry in adopting them as cohesive drug carriers [44]. For this, various combinations of storage time and temperature, after dehydration achieved by vacuum or just freezing, were studied (Table 2). NPs withstand three freezing–thaw cycles before collapsing, but maintain their monodispersity. The Z-ave and ζ-P parameters of BSA-NP, MPA@BSA-NP and RBV@BSA-NP were determined after different storage periods and conditions.

The initial colloidal suspension showed a monomodal distribution with Z-ave values of 142.33 ± 1.54 nm and ζ-P of −7.27 ± 0.80 mV on average. After the isolation and purification process, the distribution remained monomodal and the parameters changed to a Z-ave of 98.67 ± 0.80 nm and ζ-P of −17.27 ± 0.75 mV on average. These studies were also carried out with NPs with both Ass; in these cases, after freezing–thawing cycles, average Z-ave values of 108.70 ± 1.28 nm and ζ-P of −19.88 mV was obtained. The results demonstrated that centrifugation or freezing–thawing cycles can negatively affect the size of all BSA-based NPs, unlike vacuum drying, which decreased sizes and reduced the ζ-P.

NPs sometimes need chemical cross linkers to stabilize and improve their characteristics [52]. The creation of covalent connections between molecules by using these agents increases their stability and degradation resistance [36]. Cross linkers can also optimize particle size and distribution to encapsulate and release medicinal substances. To maintain NPs’ biomedical safety, the use of cross linkers must be limited because they are hazardous [52,53,54].

The findings in the stability test, contextualized by Díaz-Saldívar and Huidobro-Toro’s research [31], indicate that BSA NPs’ stability does not require a cross linker. Optimum pH and EtOH:BSA ratios ensure stability over time. The presence of AS significantly influenced the experimental size results. Previous studies indicated that introducing AS into BSA-NP produces a reduction in particle size [55,56], increasing the contact surface area between the AS and the NP.

Although, the systems designed here resulted in colloidally stable NPs with an absence of precipitation even at low ζ-P values, the physical stability of MPA@BSA-NPs and RBV@BSA-NPs cannot only be explained by electrostatic stabilization, suggesting that other factors such as hydrophobic, Van der Waals or steric overlap interactions may be responsible of this outcome [57,58].

### 3.5. Cytotoxicity Assays

The cytotoxic effects of various concentrations of free ASs or NPs were evaluated in Vero, A549 and Huh-7 mammalian cell lines. Viability percentages were determined using the MTT test after 48 h of treatment, and subsequently, CC_50_ values were calculated. Although the CC_50_ values of MPA@-BSA-NP and RBV@BSA-NP were lower than those corresponding to the empty BSA-NP, it must be noted that both AS@BSA-NPs (and consequently also the corresponding concentrations of encapsulated AS) showed highly reduced levels of cytotoxicity in comparison with both free MPA and RBV (Table 3).

Cytotoxicity studies are crucial for evaluating the biomedical application of NPs. Systematic large-scale cytotoxicity studies with polymeric NPs remain pending in the current literature, and so the use of this platform for industrial-scale applications is not yet permitted [59,60]. Since albumin constitutes approximately 50% of the proteins present in the plasma of healthy individuals and it is a multifunctional protein that can bind and transport numerous endogenous and exogenous compounds, the development of albumin-based AS transport systems is becoming more relevant in therapy against various diseases [61,62]. In fact, it must be remarked that in the three cell systems tested here, both encapsulated ASs (MPA@BSA-NP and RBV@BSA-NP) showed less cytotoxicity with higher values of CC_50_ than the free ASs. While the CC_50_ values of the free ASs were in the order of micrograms, the CC_50_ of the AS encapsulated in the AS@BSA-NPs were in the order of milligrams, demonstrating a clear improvement in this fundamental aspect.

### 3.6. Antiviral Assays

The antiviral activity of the ASs and their NPs was evaluated using mammalian cell-based viral yield reduction assays. Diverse classes of viruses were tested: the RNA ambisense JUNV, the positive-stranded RNA ZIKV, the negative-stranded RNA VSV and the double-stranded DNA virus HSV-1. Infected cultures were treated for 48 h with non-cytotoxic concentrations, after determining the AS content in the NPs considering the EʄE and the loading capacity of each one. Infected cultures treated with various concentrations of each AS or NP and infected cultures without treatment (VC) were included (Supplementary Figures S1 and S2). Treatment with BSA-NPs without an AS did not inhibit the replication of any of the four viruses in any cell line

Optimizing the biological activity of substances with a high selectivity index (SI) is more challenging than in cases where the compounds show poor activity. As can be seen in Table 4 and Table 5, the treatment of infected cultures with the two ASs and AS@BSA-NPs produced high levels of viral inhibition, but notably, out of all the cell–virus combinations evaluated here, the AS@BSA-NPs showed a higher SI than the free ASs (Table 4 and Table 5). It is noteworthy that the IC_50_ values of the free ASs were between 1 and 5 orders of magnitude higher than those obtained with encapsulated AS, while the IS values were between 1 and 8 orders of magnitude higher for AS-BSA-NPs compared to free ASs.

Taking into consideration the influence of the host cell, for free MPA no great differences were observed in the SI of the four viruses in all the tested cell types. But, the antiviral effectiveness of MPA@BSA-NP was always greater than MPA with variations depending on the host cell. For JUNV and ZIKV, 100–1000-fold-lower SIs were obtained when the tests were performed in Vero cells in comparison to A549 and Huh-7 cells. In contrast, the SI were similar for HSV-1 and VSV in the two cell lines tested, Vero and A549 (Table 4). Regarding free RBV, no important differences were detected for each virus in the three cell lines tested. In the case of RBV@BSA-NP, the SI was lower in Vero cells for all viruses analyzed, but with variations according to the virus and host cell (Table 5). In particular, treatment with both MPA@BSA-NP and RBV@BSA-NP showed greater viral inhibition in Huh-7 cells, with IC_50_ values often one to three orders of magnitude lower than in Vero and A549 cells. This could be due to the high metabolic rate of Huh-7 cells as they are of hepatic origin, suggesting that the possible influence of the cell tissue in the enhancement of the antiviral effect of AS@BSA-NPs may be further investigated.

In addition to our previous findings [35], the present data reveal that this alternative BSA-based delivery strategy for encapsulating MPA or RBV also achieves a reduction in the concentration of AS required to assess a significant antiviral effect, as reflected by the high SI obtained in all evaluated cases. Additionally, we calculated the SI using the total amount of AS@BSA-NPs (Supplementary Tables S1 and S2). When analyzing the results, it is outstanding to consider that the effective concentration of AS was nearly 200 and 100-fold lower in MPA@BSA-NP and RBV@BSA-NP, respectively, compared to the free forms.

MPA and RBV have demonstrated efficacy against a variety of RNA and DNA viruses [16,63,64], but this is the first report on their antiviral activity against the rhabdovirus VSV. Since MPA and RBV’s in vivo efficacy can be limited by factors such as poor bioavailability, systemic toxicity and rapid clearance, nanotechnology offers a promising approach to enhance drug delivery and improve therapeutic outcomes. By encapsulating MPA and RBV within nanosystems, such as those based on BSA, it is possible to protect the ASs from degradation, increase their cellular uptake and target them to specific tissues. Thus, our results significantly expand the known antiviral spectrum of these molecules and highlight the potential of BSA NPs as a novel drug delivery strategy to enhance antiviral efficacy minimizing adverse effects.

## 4. Conclusions

The results obtained here demonstrate that the BSA NP prepared by the desolvation method present more effective antiviral activity against JUNV, ZIKV, HSV-1 and VSV in comparison with free compounds, revealing a versatile platform to optimize different biological requirements. The encapsulation of active principles in BSA NPs using the desolvation method appears to be a potentially efficient system for the delivery of ASs. This is reflected in the high SIs and the low amounts of the active substance used in the cellular assays. Our results demonstrate antiviral effects across all four viruses tested in different host cells, underscoring the potential of this drug delivery system in antiviral therapy.

## Figures and Tables

**Table 1:** Characterization of colloidal and purified NP suspensions.

Sample	Z-Ave * (d. nm)	PdI	ζ-P (mV)
Colloidal NP suspensions			
BSA-NP	155.5 ± 1.30	0.171	−6.25 ± 1.05
MPA@BSA-NP	152.4 ± 1.59	0.215	−7.56 ± 0.47
RBV@BSA-NP	120.4 ± 1.74 *	0.156	−8.00 ± 0.89
Purified NP			
BSA-NP	74.3 ± 2.7	0.243	−17.54 ± 0.60
MPA@BSA-NP	59.6 ± 8.4 *	0.270	−16.97 ± 0.45
RBV@BSA-NP	56.1± 10.9 **	0.289	−17.17 ± 0.65

Results are expressed as the mean ± SD (n = 6). *p*-values were determined using an ANOVA analysis, followed by Dunnett’s post hoc test. * Represents significant differences vs. each BSA-NP; * *p* < 0.05, ** *p* < 0.01.

**Table 2:** NP stability evaluation.

Conditions	Sample	Z-Ave * (d. nm)	PdI	ζ-P * (mV)
Colloidal suspension	BSA-NP	155.5 ± 1.30	0.171	−6.25 ± 1.05
	MPA@BSA-NP	152.4 ± 1.59	0.215	−7.56 ± 0.47
	RBV@BSA-NP	120.4 ± 1.74 *	0.156	−8.00 ± 0.89
Colloidal suspension	BSA-NP	255.4 ± 2.2	0.408	−14.88 ± 6.10 *
stored at 25 °C	MPA@BSA-NP	207.5 ± 4.4 **	0.243	−9.05 ± 0.71
(3 months)	RBV@BSA-NP	238.7 ± 5.6 *	0.395	−9.21 ± 0.18
Purified NP	BSA-NP	139.1 ± 0.95	0.23	−17.54 ± 0.60
	MPA@BSA-NP	86.7 ± 0.59 **	0.27	−16.97 ± 0.45
	RBV@BSA-NP	70.81 ± 0.86 **	0.289	−17.17 ± 0.65
NP	BSA-NP	131.3 ± 1.95	0.366	−20.78 ± 0.16
(freezing/thaw)	MPA@BSA-NP	101.9 ± 0.72 **	0.182	−17.83 ± 0.32
	RBV@BSA-NP	92.91 ± 1.16 ***	0.219	−21.03 ± 0.40
NP	BSA-NP	90.65 ± 5.28	0.402	−6.51 ± 0.25
Vacuum drying	MPA@BSA-NP	72.45 ± 0.78 **	0.493	−12.6 ± 0.36 *
	RBV@BSA-NP	71.29 ± 2.69 **	0.608	−6.62 ± 2.05
NP	BSA-NP	142.2 ± 9.17	0.586	−5.99 ± 0.45
stored −80 °C	MPA@BSA-NP	104.9 ± 3.56 **	0.612	−6.8 ± 0.93
(3 months)	RBV@BSA-NP	96.72 ± 2.69 ***	0.442	−6.8 ± 0.53
NP	BSA-NP	154.8 ± 3.2	0.374	−15.8 ± 1.83 *
stored at 25 °C	MPA@BSA-NP	124.7 ± 8.2 **	0.245	−21.43 ± 0.68
(3 months)	RBV@BSA-NP	139.4 ± 0.9 *	0.277	−17.53 ± 1.59

Results are expressed as the mean ± SD (n = 6). *p*-values were determined using an ANOVA analysis, followed by Dunnett’s post hoc test. * Represents significant differences versus BSA-NP, * *p* < 0.05, ** *p* < 0.01, *** *p*< 0.001.

**Table 3:** Cytotoxicity.

CC_50_ [mg/mL]							
Cell Line	BSA-NP	MPA	MPA@BSA-NP		RBV	RBV@BSA-NP	
			MPA@BSA-NP	MPA		RBV@BSA-NP	RBV
*Vero*	821.10 ± 26.25	0.16 ± 0.46	522.64 ± 31.65	34.5 ± 2.4	0.09 ± 0.32	295.58 ± 21.31	0.30 ± 0.45
*A549*	812.10 ± 10.25	0.16 ± 0.46	582.64 ± 11.65	38.5 ± 3.6	0.09 ± 0.32	305.28 ± 10.84	0.31 ± 0.75
*Huh-7*	800.10 ± 30.51	0.16 ± 0.46	502.64 ± 10.65	33.2 ± 1.6	0.09 ± 0.32	325.28 ± 20.84	0.33 ± 0.63

CC_50_, free compound or NP concentration required to reduce cell viability by 50% after 48 h of incubation. The quantity of AS in AS@BSA-NPs was calculated using the EʄE, the values are presented in the table adjacent to the AS@BSA-NP concentration. Results are expressed as the mean ± SD (n = 3).

**Table 4:** Antiviral activity of MPA vs. MPA in MPA@BSA-NP.

Virus	Treatment	Vero		A549		Huh-7	
		IC_50_ [ng/mL]	SI	IC_50_ [ng/mL]	SI	IC_50_ [ng/mL]	SI
JUNV	MPA	208.22 ± 2.12	7.7 × 10^2^	160.17 ± 40.21	1.0 × 10^3^	124.93 ± 2.01	1.0 × 10^3^
	MPA@BSA-NP	64.16 ± 42 **	5.4 × 10^5^	34.16 ± 22 **	1.1 × 10^6^	0.078 ± 0.31 ***	4.3 × 10^8^
ZIKV	MPA	294.71 ± 1.25	5.4 × 10^2^	285.10 ± 5.63	5.6 × 10^2^	160.17 ± 5.26	1.0 × 10^3^
	MPA@BSA-NP	0.18 ± 0.01 ***	1.9 × 10^8^	0.05 ± 0.02 ***	7.7 × 10^8^	0.02 ± 0.012 ***	1.7 × 10^9^
HSV-1	MPA	256.27 ± 40.21	6.2 × 10^2^	101.63 ± 0.009	1.6 × 10^3^	ND	ND
	MPA@BSA-NP	0.002 ± 0.003 ***	1.7 × 10^10^	0.001 ± 0.009 ***	3.9 × 10^10^	ND	ND
VSV	MPA	320.34 ± 39.66	4.9 × 10^2^	240.26 ± 22.47	6.7 × 10^2^	ND	ND
	MPA@BSA-NP	0.0012 ± 0.001 ***	2.9 × 10^10^	0.0006 ± 0.005 ***	6.4 × 10^10^	ND	ND

IC_50_: compound concentration required to reduce viral yield by 50%, determined by plaque assay. SI: rate CC_50_/IC_50_. The quantity of MPA in MPA@BSA-NP was determined using the EʄE. The values in the table represent the quantity of MPA contained within the MPA@BSA-NPs. Results are expressed as the mean ± SD (n = 3). *p*-values analysis of variance (ANOVA) with Dunnett’s post hoc test vs. versus MPA. Represents significant differences versus MPA, ** *p* < 0.01, *** *p* < 0.001. ND = not done.

**Table 5:** Antiviral activity of RBV vs. RBV in RBV@BSA-NP.

Virus	Treatment	Vero		A549		Huh-7	
		IC_50_ [ng/mL]	SI	IC_50_ [ng/mL]	SI	IC_50_ [ng/mL]	SI
JUNV	RBV	7822.85 ± 951.52	1.2 × 10^1^	6372.45 ± 955.05	1.4 × 10^1^	4623.15 ± 572.26	1.9 × 10^1^
	RBV@BSA-NP	4.80 ± 2.23 ***	6.3 × 10^4^	1.25 ± 1.30 ***	2.5 × 10^5^	0.036 ± 0.04 ***	9.2 × 10^6^
ZIKV	RBV	306.25 ± 2.68	2.9 × 10^2^	117.62 ± 1.56	7.6 × 10^2^	93.13 ± 2.56	9.7 × 10^2^
	RBV@BSA-NP	8.44 ± 10.25 **	3.6 × 10^4^	0.835 ± 0.51 ***	3.7 × 10^5^	0.11 ± 0.15 ***	3.0 × 10^6^
HSV-1	RBV	2450 ± 231	1.6 × 10^2^	2050 ± 125	1.9 × 10^2^	ND	ND
	RBV@BSA-NP	0.11 ± 0.16 ***	2.7 × 10^6^	0.06 ± 0.2 ***	5.2 × 10^6^	ND	ND
VSV	RBV	3200.34 ± 126	1.2 × 10^2^	1800 ± 189	2.2 × 10^2^	ND	ND
	RBV@BSA-NP	31.97 ± 0.43 **	9.4 × 10^3^	15.98 ± 0.24 **	1.9 × 10^4^	ND	ND

IC_50_: compound concentration required to reduce viral yield by 50%, determined by plaque assay. SI: rate CC_5_0/IC_50_. The quantity of RBV in the RBV@BSA-NPs was determined using EʄE. The values in the table represent the quantity of RBV contained within the RBV-BSA-NPs. Results are expressed as the mean ± SD (n = 3). *p*-values analysis of variance (ANOVA) with Dunnett’s *post hoc* test vs. MPA. Represents significant differences versus RBV, ** *p* < 0.01, *** *p* < 0.001. ND = not done.

## Data Availability

The data that support the findings of this study are available from the corresponding author upon reasonable request.

## Supplementary Materials

The following supporting information can be downloaded at: https://www.mdpi.com/article/10.3390/v17020138/s1, Figure S1: Antiviral effect.; Figure S2: Antiviral effect; Table S1: Antiviral activity of MPA vs MPA@BSA-NP; Table S2: Antiviral activity of RBV vs RBV@BSA-NP.
