# Supplementary material for: Bovine Serum Albumin Nanoparticle-Mediated Delivery of Ribavirin and Mycophenolic Acid for Enhanced Antiviral Therapeutics
Source: Viruses. 2025 Jan 21;17(2):138. doi: 10.3390/v17020138 (PMC11860702; doi:10.3390/v17020138)
Supplement: Supplementary file 1 [file viruses-17-00138-s001.zip › viruses-3373700-supplementary.pdf]

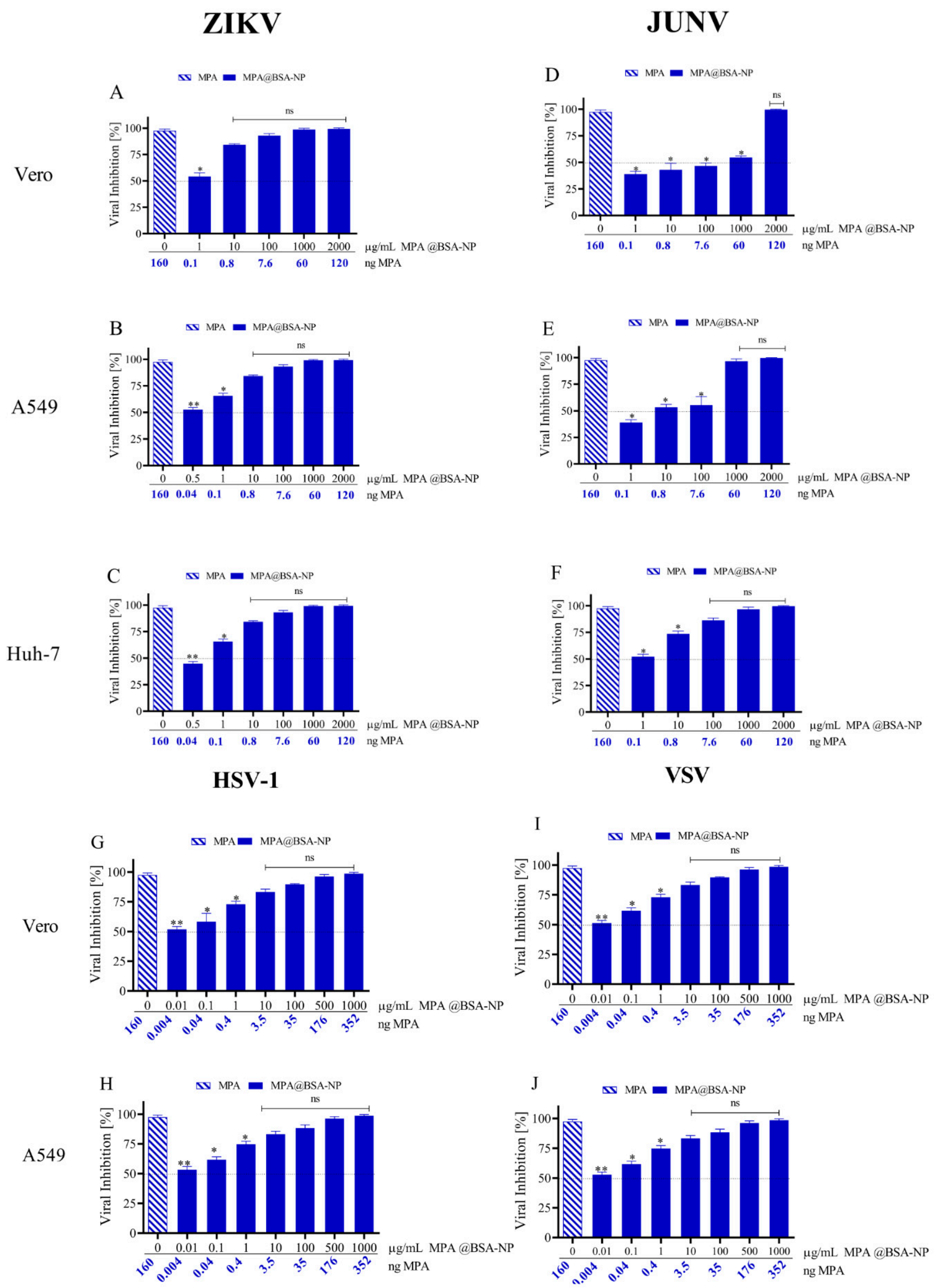

Supplementary Figure S1. Antiviral effect. Vero, A549 and Huh-7 cells were infected with ZIKV (MOI 0.1) (A, B and C), JUNV (MOI 0.1) (D, E and F), HSV-1 (MOI 0.07) (G and H), and VSV (MOI 0.07) (I and J), after 1 h of adsorption at 37 °C the inocula were removed and cells were incubated in the absence (VC) or presence of 160 ng/mL of free MPA or different concentrations of MPA@BSA-NP. At 48 h p.i., supernatant cultures were harvested, and extracellular virus yields were determined by a plaque assay. In the x-axis the concentration of MPA@BSA-NP and the corresponding MPA encapsulated (calculated according to EƒE) are presented for each point. Results are expressed as the mean ± SD (n = 3). *p*-values were determined using an ANOVA analysis, followed by Dunnett's *post hoc* test. * Represents significant differences respect to MPA; * $p < 0.05$, ** $p < 0.01$, *** $p < 0.001$.

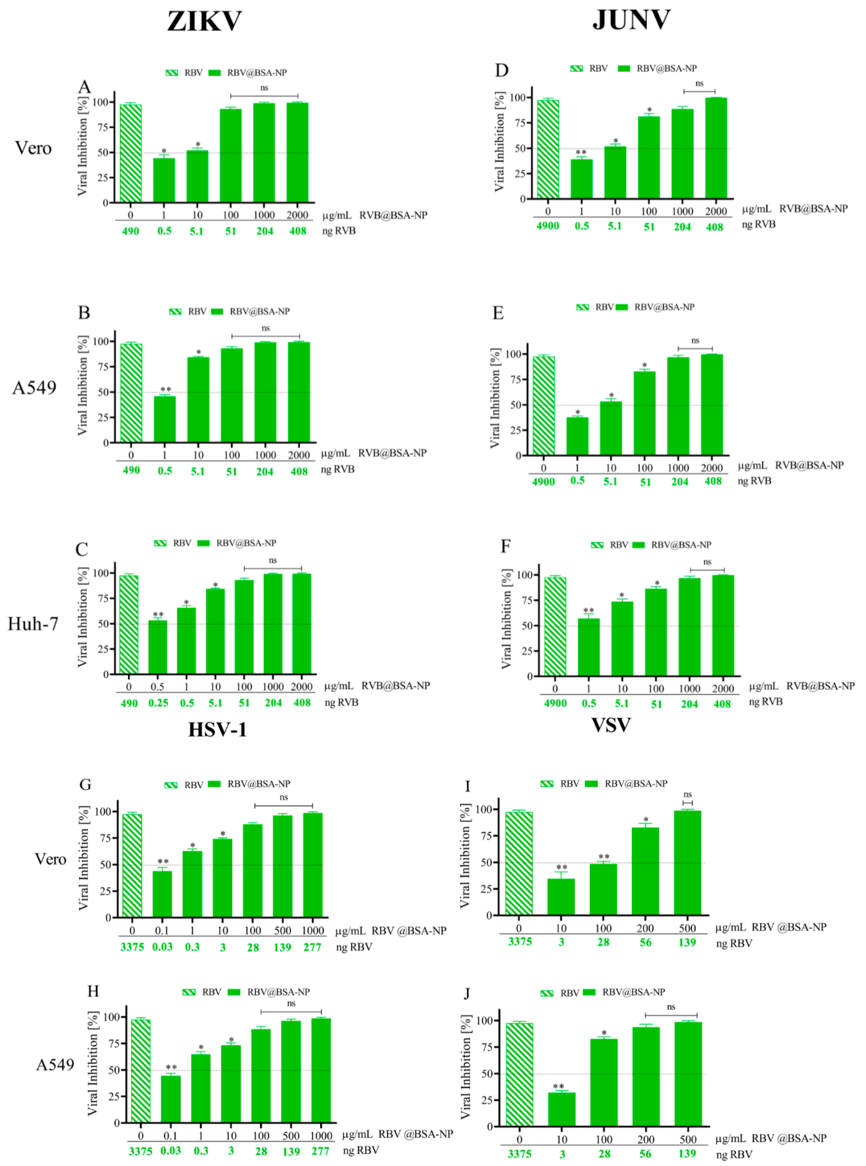

Supplementary Figure S2. Antiviral effect. Vero, A549 and Huh-7 cells were infected with ZIKV (MOI 0.1) (A, B and C), JUNV (MOI 0.1) (D, E and F), HSV-1 (MOI 0.07) (G and H), and VSV (MOI 0.07) (I and J), and after 1 h of adsorption at 37 °C the inocula were removed and cells were incubated in the absence (VC) or presence of 490 ng/mL of RBV for ZIKV, 4900 ng/mL of RBV for JUNV, and 3375 ng/mL of RBV for HSV-1 and VSV or different concentrations of RBV@BSA-NP. At 48 h p.i., supernatant cultures were harvested, and extracellular virus yields were determined by a plaque assay. In the x-axis the concentration of RBV@BSA-NP and the corresponding concentration of RBV encapsulated (calculated according to E∫E) are presented for each point. Results are expressed as the mean ± SD (n = 3). *p*-values were determined using an ANOVA analysis, followed by Dunnett's *post hoc* test. * Represents significant differences respect to RBV; * $p < 0.05$, ** $p < 0.01$, *** $p < 0.001$.

Supplementary Table S1. Antiviral activity of MPA *vs* MPA@BSA-NP

Virus	Treatment	Vero		A549		Huh-7	
		IC_{50} [μg/mL]	SI	IC_{50} [μg/mL]	SI	IC_{50} [μg/mL]	SI
JUNV	MPA	0.21 ± 0.12	7.6 10^2	0.16 ± 0.21	1.0 10^3	0.12 ± 0.01	1.3 10^3
	MPA@BSA-NP	10.52 ±1.4**	5.0 10^4	8.49 ± 2.5**	6.9 10^4	1.2 ± 0.8**	4.2 10^5
ZIKV	MPA	0.29 ± 0.25	5.5 10^2	0.29 ± 0.63	5.5 10^2	0.16 ±0.26	1.0 10^3
	MPA@BSA-NP	1.46 ± 0.5***	3.6 10^5	0.45 ± 0.03*	1.3 10^6	0.23 ± 0.06	2.2 10^6
HSV-1	MPA	0.26 ± 0.21	6.2 10^2	0.10 ± 0.009	1.6 103	ND	ND
	MPA@BSA-NP	0.02 ± 0.07*	2.6 10^7	0.01 ± 0.09**	5.8 10^7	ND	ND
VSV	MPA	0.32 ± 0.66	5.0 10^2	0.24 ± 0.47	6.7 10^2	ND	ND
	MPA@BSA-NP	0.11 ± 0.2*	4.8 10^6	0.01 ± 0.3*	5.8 10^7	ND	ND

IC_{50}: compound concentration required to reduce viral yield by 50%, determined by plaque assay. SI: rate CC_{50}/IC_{50}. The SI was calculated using the CC_{50} of MPA@BSA-NPs. Results are expressed as the mean ± SD (n = 3). *p*-values analysis of variance (ANOVA) with Dunnett's *post hoc* test vs versus MPA. *Represents significant differences versus MPA, ***p* < 0.01, ****p* < 0.001. ND = not done.

Supplementary Table S2. Antiviral activity of RBV *vs* RBV@BSA-NP

Virus	Treatment	Vero		A549		Huh-7	
		IC_{50} [μg/mL]	SI	IC_{50} [μg/mL]	SI	IC_{50} [μg/mL]	SI
JUNV	RBV	7.82 ± 0.95	12	6.37 ± 1.25	14	4.62 ± 0.2	19
	RBV@BSA-NP	10.12 ± 2.08*	2.9 10^4	5.45 ± 1.9	5.6 10^4	1.42 ± 0.52*	2.3 10^5
ZIKV	RBV	0.31 ± 0.2	294	0.12 ± 0.15	7.5 10^2	0.09 ± 0.05	1.0 10^3
	RBV@BSA-NP	8.5 ± 1.30**	3.5 10^4	1 ± 0.1*	3.1 10^5	0.5 ± 0.01*	6.5 10^5
HSV-1	RBV	2.45 ± 0.23	37	2.08 ± 0.12	43	ND	ND
	RBV@BSA-NP	1.48 ± 0.05*	2.0 10^5	0.1 ± 0.03*	3.1 10^6	ND	ND
VSV	RBV	3.20 ± 0.12	28	1.8 ± 0.18	50	ND	ND
	RBV@BSA-NP	98.5 ± 4.52***	3.0 10^3	14.5 ± 2.31**	2.1 10^4	ND	ND

IC_{50}: compound concentration required to reduce viral yield by 50%, determined by plaque assay. SI: rate CC_{50}/IC_{50}. The SI was calculated using the CC_{50} of RBV@BSA-NPs. Results are expressed as the mean ± SD (n = 3). *p*-values analysis of variance (ANOVA) with Dunnett's *post hoc* test vs versus MPA. *Represents significant differences versus MPA, ***p* < 0.01, ****p* < 0.001. ND = not done.
